# Radiomics-Based Characterization of Aggressive Prostate Cancer Variants: Diagnostic Challenges and Opportunities

**DOI:** 10.3390/cancers18050780

**Published:** 2026-02-28

**Authors:** Katarzyna Sklinda, Martyna Rajca, Marek Kasprowicz, Łukasz Michałowski, Michał Małek, Bartłomiej Olczak, Jerzy Walecki

**Affiliations:** 1 Centre of Radiological Diagnostics, National Medical Institute of the Ministry of the Interior and Administration, Wołoska 137, 02-507 Warsaw, Poland; katarzyna.sklinda@pimmswia.gov.pl (K.S.);; 2 Clinical Department of Urology and Oncological Urology, National Medical Institute of the Ministry of the Interior and Administration, Wołoska 137, 02-507 Warsaw, Poland; 3 Department of Pathology, Medical University of Warsaw, Pawińskiego, 02-106 Warsaw, Poland; 4 Department of Radiology, Centre of Postgraduate Medical Education, Marymoncka 99/103, 01-813 Warsaw, Poland

**Keywords:** aggressive prostate cancer variants, review, radiomics, multiparametric MRI, imaging biomarkers, tumor heterogeneity, neuroendocrine prostate cancer, ductal adenocarcinoma, small cell carcinoma, Gleason pattern 5

## Abstract

Background/Objectives: Aggressive variants of prostate cancer pose significant diagnostic and prognostic challenges due to atypical imaging appearances, variable prostate-specific antigen behavior, and distinct molecular features. Conventional imaging may underestimate their biological aggressiveness. This review aimed to synthesize current evidence on imaging characteristics, biomarker dynamics, tumor localization, histology, and radiomic features of aggressive prostate cancer variants, and to evaluate the potential role of radiomics in early recognition and risk stratification. Methods: A structured narrative review was performed of studies reporting imaging, clinical, and molecular features of aggressive prostate cancer variants. Imaging modalities included multiparametric magnetic resonance imaging, positron emission tomography with prostate-specific membrane antigen or fluorodeoxyglucose, bone scintigraphy, and transrectal ultrasound. Data on prostate-specific antigen levels and kinetics, intraprostatic tumor location, tumor size, metastatic patterns, and molecular alterations were extracted. Evidence for rare entities such as basaloid and primary squamous carcinomas was derived from published case reports and series, while selected variants were complemented by institutional imaging and histopathologic observations. Results: Neuroendocrine and small cell carcinomas frequently showed low prostate-specific antigen levels, high fluorodeoxyglucose uptake, low prostate-specific membrane antigen expression, and central or transitional zone involvement with large tumor size at diagnosis. Ductal adenocarcinoma demonstrated marked diffusion restriction and elevated prostate-specific antigen, whereas basal cell carcinoma often appeared inconspicuous on conventional imaging. Radiomic analysis consistently captured tumor heterogeneity and spatial complexity beyond standard qualitative metrics. Conclusions: Aggressive prostate cancer variants represent a diagnostic blind spot in routine imaging. Radiomics offers complementary quantitative information that may improve early detection, subtype differentiation, and risk stratification when integrated into multimodal imaging workflows. Further prospective and radiogenomic studies are warranted to validate these findings.

## Simple Summary

Aggressive forms of prostate cancer are uncommon but clinically important because they often behave differently from typical prostate cancer and can be missed by standard imaging and blood tests. Some of these tumors produce little or no prostate-specific antigen and may appear less suspicious on routine scans, despite being biologically aggressive. This review aims to clarify how advanced imaging techniques, together with radiomics—an approach that extracts quantitative information from medical images—can help identify these high-risk cancer subtypes earlier and more accurately. By summarizing current evidence on imaging appearance, tumor location, biomarker behavior, and underlying biology, this work highlights where conventional assessment may fail and how radiomics can add clinically useful information. These findings may support better risk stratification, guide personalized treatment decisions, and inform future research on aggressive prostate cancer variants.

## 1. Introduction

Prostate cancer remains the most frequently diagnosed non-cutaneous malignancy and the second leading cause of cancer-related death among men worldwide [1]. While most prostate cancers follow an indolent course and are detected early via PSA screening and imaging, a distinct subset of tumors exhibits aggressive behavior, rapid progression, and poor clinical outcomes. These aggressive variants of prostate cancer represent a diagnostic and therapeutic challenge due to their divergent histologic and molecular characteristics.

Among these variants, the most clinically relevant include neuroendocrine prostate cancer (NEPC), small cell carcinoma, ductal adenocarcinoma, high-grade acinar adenocarcinoma with Gleason pattern 5, and the rare basaloid or squamous subtypes. Although their cumulative incidence is relatively low—estimated at approximately 5–10% of all prostate cancers—these entities account for a disproportionate number of prostate cancer–related deaths, due to their frequent presentation at advanced stages and resistance to standard androgen deprivation therapy [2,3].

NEPC and small cell carcinoma, whether de novo or arising as treatment-emergent forms after androgen-targeted therapy, represent less than 2% of newly diagnosed cases but may occur in 10–20% of patients with metastatic castration-resistant prostate cancer (mCRPC) [4,5].

Ductal adenocarcinoma constitutes approximately 0.4–0.8% of prostate cancers in population registries, although its frequency may be underestimated due to diagnostic overlap with acinar patterns [6].

Gleason pattern 5 tumors, although not a distinct histologic subtype, represent the most aggressive end of the acinar spectrum and are present in approximately 15–20% of patients undergoing radical prostatectomy in high-risk cohorts [7].

Basaloid and squamous carcinomas of the prostate are exceedingly rare, with only case reports and small series available, but are important due to their atypical behavior and lack of response to conventional therapies [2].

These tumors often evade early detection due to their non-classical PSA kinetics, atypical MRI features, and, in many cases, low or absent PSMA expression. As such, a nuanced understanding of their radiologic appearance, clinical presentation, and molecular background is critical for timely diagnosis and effective management. Recent advances in multiparametric MRI, PSMA and FDG PET/CT, and radiomics-based image analysis offer promising opportunities to improve non-invasive identification of these subtypes.

Recent reviews on prostate cancer imaging have primarily been structured by modality (mpMRI, PSMA PET/CT, FDG PET/CT) or by major clinical indications (primary diagnosis, biochemical recurrence, staging), and therefore often under-emphasize rare but clinically consequential variant histologies and aggressive phenotypes that do not follow canonical PSA kinetics or PSMA-expression patterns. In contrast, the present review is explicitly organized around aggressive variant-associated diagnostic blind spots, integrating imaging appearance with PSA behaviour, tumour topography, and molecular context to support recognition of biologically aggressive disease even when routine imaging algorithms or single-metric interpretations are misleading. This approach provides a clinically oriented framework that links multi-modal imaging findings to decision-relevant endpoints (local extension, metastatic risk, and phenotype-dedifferentiation cues), rather than offering a modality-centric catalogue of features [8,9,10,11].

In this review, we present a comprehensive analysis of the imaging characteristics, clinical features, and molecular biology of aggressive prostate cancer variants. Special emphasis is placed on quantitative imaging (radiomics/AI) and clinically discordant scenarios that represent diagnostic pitfalls in aggressive variants. We also explore the diagnostic potential of radiomic profiling and its integration into precision oncology workflows.

## 2. Methods

### 2.1. Study Design and Scope

We performed a structured narrative review to characterize the imaging, clinical, and molecular characteristics of aggressive prostate cancer variants. The analysis focused on neuroendocrine prostate cancer (NEPC), small cell carcinoma, ductal adenocarcinoma, high-grade acinar adenocarcinoma with predominant Gleason pattern 5 (Grade Group 5), and the rare basaloid (basal cell) and primary squamous subtypes. The objective was to synthesize available evidence and integrate representative imaging examples to highlight subtype-specific diagnostic patterns and clinically relevant imaging pitfalls.

Published data were complemented with institutional cases, including original multiparametric MRI (mpMRI) and histopathologic images of small cell carcinoma, high-grade acinar adenocarcinoma (Gleason score 5 + 5), and ductal adenocarcinoma. As no cases of prostatic basal cell carcinoma or primary squamous cell carcinoma were identified at our institution, these entities were characterized exclusively using published reports.

### 2.2. Data Sources and Study Selection

Eligible studies included retrospective cohorts, imaging series, case series, and case reports reporting at least one of the predefined aggressive variants. Studies were required to provide clinical, imaging, or molecular data relevant to tumor characterization. Narrative reviews without original patient data were excluded from formal synthesis but were consulted for contextual background.

For basaloid carcinoma and primary squamous cell carcinoma, inclusion was restricted to PubMed/MEDLINE-indexed articles published between 2015 and 2025 reporting primary human cases. This search yielded eight publications describing 17 patients with basaloid carcinoma [12,13,14,15,16,17,18,19] and five publications describing five patients with primary squamous cell carcinoma [20,21,22,23,24].

### 2.3. Data Extraction and Synthesis

Extracted variables included MRI features (T2-weighted signal characteristics, diffusion restriction, apparent diffusion coefficient values, enhancement patterns), PET tracer uptake (PSMA and FDG), PSA levels and kinetics, anatomical tumor distribution (central/periurethral vs. peripheral), tumor size at diagnosis, metastatic patterns, and reported molecular alterations.

A qualitative synthesis was performed to identify recurring imaging phenotypes, diagnostic challenges, and biologically meaningful patterns across variants. Imaging findings from mpMRI, PSMA PET/CT, FDG PET/CT, transrectal ultrasound, and bone scintigraphy were integrated to describe subtype-specific imaging behavior. Molecular and immunohistochemical data (including TP53, RB1, PTEN, MYCN, AURKA, SPOP, and CK5/6 expression) were extracted when available and interpreted alongside imaging features.

Radiomics and quantitative imaging studies were reviewed to contextualize the potential role of texture-based features, kurtosis metrics, and ADC-derived parameters in differentiating aggressive histologic subtypes.

### 2.4. Methodological Limitations

The rarity of basaloid and primary squamous carcinomas limits sample size and generalizability and necessitates reliance on retrospective case reports without institutional validation. Across all variants, the included studies exhibited heterogeneity in imaging protocols, reporting standards, and molecular characterization. Additionally, most imaging and radiomics studies were retrospective and lacked external validation or multicenter design, particularly for rare histologic subtypes. These limitations were considered when interpreting the synthesized findings.

## 3. Overview of Aggressive Prostate Cancer Variants

**Figure 1:**
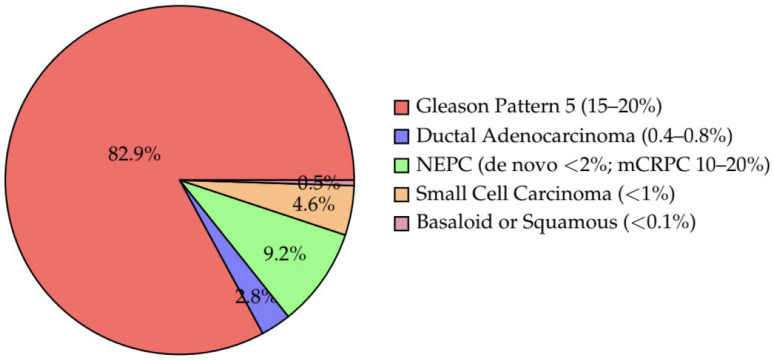
Estimated Frequency of Aggressive Prostate Cancer Variants.

The WHO 2022 classification of prostate tumors recognizes several histological variants beyond conventional acinar adenocarcinoma, some of which are associated with highly aggressive clinical behavior and a poor prognosis [25]. These aggressive subtypes are rare but clinically significant, as they often present atypically, progress rapidly, and exhibit distinct imaging and molecular features. The estimated frequency of aggressive prostate cancer variants is presented in Figure 1. This review focuses on five key aggressive prostate cancer variants: neuroendocrine prostate cancer (NEPC), ductal adenocarcinoma, small cell carcinoma, high-grade acinar adenocarcinoma with predominant Gleason pattern 5 and basaloid/squamous carcinoma.

Neuroendocrine prostate cancer (NEPC) and small cell carcinoma are defined by early visceral metastases, minimal or absent production of prostate-specific antigen (PSA), and lack of activity of the androgen receptor (AR) pathway. These subtypes are often treated emergently, arising after long-term androgen deprivation therapy (ADT) as a mechanism of lineage plasticity. They are genomically characterized by mutations in TP53, RB1, AURKA, and MYCN, and typically show loss of PSMA expression while exhibiting high uptake on FDG PET/CT, complicating conventional imaging approaches [5,26].

Ductal adenocarcinoma, first described as a variant with endometrioid features, commonly occurs near the prostatic urethra and can present with obstructive urinary symptoms or hematuria. It tends to have a rapid PSA velocity and often invades the seminal vesicles. Despite the expression of AR, PSA, and PSMA, its clinical behavior is significantly more aggressive than that of acinar carcinoma. Ductal tumors frequently harbor ERG gene fusions, SPOP mutations, and TP53 alterations [4,27].

High-grade acinar adenocarcinoma with a predominant Gleason pattern 5, including comedo and solid architectural types, is not recognized as a separate histologic variant, but it is critical from a prognostic point of view. These tumors show extensive undifferentiated growth, are more likely to extend beyond the prostate capsule, and often exhibit lymphovascular invasion. Molecularly, these tumors are frequently associated with alterations in the PTEN, TP53, and SPOP genes, and show strong uptake in both PSMA and FDG PET/CT [2,28].

Basal cell carcinoma of the prostate (basaloid/adenoid-cystic subtype) is an exceptionally rare entity (≈0.01% of prostate cancers) and biologically distinct from acinar adenocarcinoma, with reports describing local aggressiveness and metastatic potential [12,13]. A review of PubMed/MEDLINE articles published between 2015 and 2025 identified 8 publications describing 17 individual patients: Simper et al. (9 cases), Hennes et al. (1 case), Dong et al. (1 case), Ridai et al. (1 case), Wang et al. (1 case), Low et al. (2 cases), Taskovska et al. (1 case), and Toesca et al. (1 case) [12,13,14,15,16,17,18,19]. Genomic profiling highlights PTEN loss, EGFR overexpression, and recurrent copy-number loss of chromosome 16 with candidate drivers (e.g., CYLD), underscoring divergence from conventional prostate adenocarcinoma [13,15,17].

Primary squamous cell carcinoma of the prostate is also extremely rare (well below 1% of prostate malignancies) and biologically aggressive, with reports of early visceral and osseous metastases and a median survival of approximately one year in historical series [20]. Over the same 10-year window, 6 case-bearing publications were identified, each reporting a single patient [20,21,22,23,24,29].

Across all these aggressive histologic variants, common clinical and biological hallmarks include rapid progression, resistance to conventional therapies, atypical radiologic appearances, low or absent PSA expression, and early visceral metastasis, as summarized in Table 1. Recurrent molecular alterations—including TP53, RB1, PTEN, and MYCN—contribute to their aggressive phenotype and represent potential targets for molecular diagnostics and precision therapeutics [30].

## 4. Imaging Characteristics of Aggressive Variants

### 4.1. Multiparametric MR

Multiparametric magnetic resonance imaging (mpMRI), which integrates T2-weighted imaging (T2WI), diffusion-weighted imaging (DWI), dynamic contrast-enhanced imaging (DCE), and optionally MR spectroscopy, remains the primary non-invasive modality for prostate cancer diagnosis and localization. However, aggressive histologic variants frequently display atypical imaging characteristics that fall outside PIRADS v2.1 criteria and pose diagnostic challenges [31] (Table 2).

Neuroendocrine prostate cancer (NEPC) and small cell carcinoma often present as large, infiltrative masses within the central or transitional zones, characterized by marked T2 hypointensity and significantly restricted diffusion, reflected in very low ADC values. Quantitative ADC analysis in these variants demonstrates both lower mean values and reduced kurtosis, indicating uniform cellular density and aggressive growth patterns [32]. Semi-quantitative approaches, such as histogram-based metrics and advanced texture analysis, may further assist in differentiating NEPC from conventional acinar tumors.

**Figure 2:**
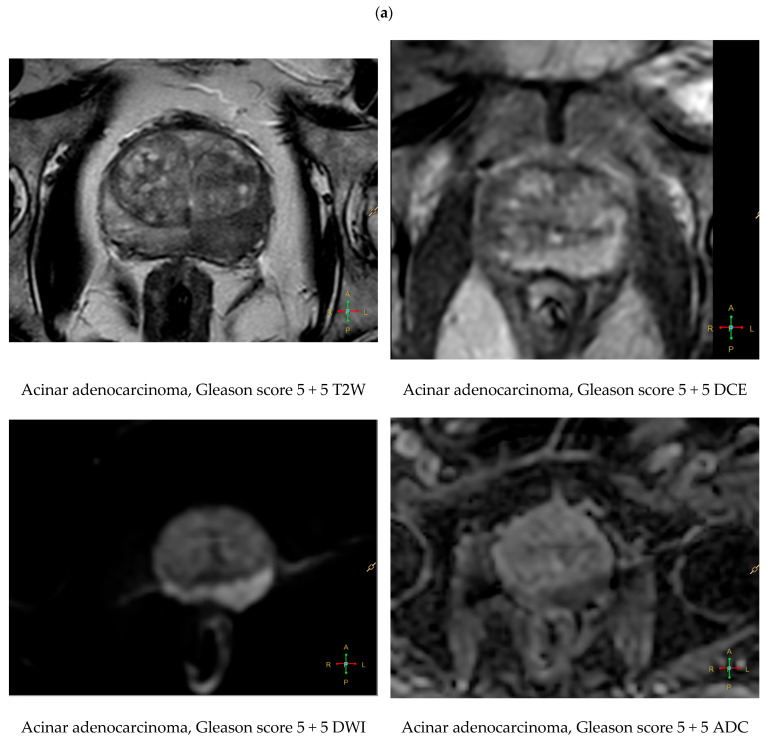

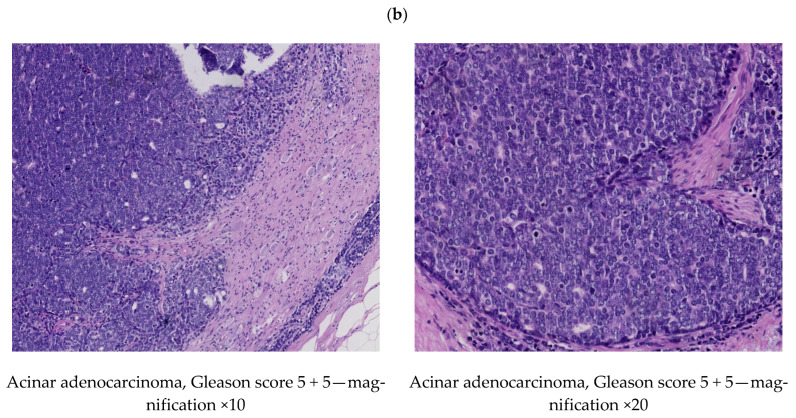
(**a**) Multiparametric MRI of high-grade acinar prostate adenocarcinoma (Gleason score 5 + 5, Grade Group 5) in the peripheral zone of the left lobe. On axial T2-weighted imaging, the lesion appears as a markedly hypointense, ill-defined focus with distortion of normal zonal anatomy. Diffusion-weighted imaging (DWI) demonstrates very high signal intensity with markedly reduced apparent diffusion coefficient (ADC) values, while dynamic contrast-enhanced (DCE) imaging shows early and intense enhancement. These findings are consistent with aggressive tumor biology and correspond to PIRADS 5. (**b**) Histological image of prostate acinar adenocarcinoma, Gleason score 5 + 5 (Grade Group 5). The tumor exhibits undifferentiated solid growth with loss of glandular architecture, characteristic of highly aggressive phenotype. Hematoxylin and eosin (H&E) staining. Representative fields shown at ×10 (**left**—perineuronal invasion) and ×20 magnification (**right**—extraprostatic extension and invasion into the prostatic neuronal plexus).

High-grade acinar adenocarcinomas with Gleason pattern 5 frequently exhibit multifocal PIRADS 5 lesions with severe T2 hypointensity and very low ADC values, especially in the peripheral zone (Figure 2). Spectroscopic MRI may reveal elevated choline and reduced citrate concentrations in aggressive tumors, aiding in metabolic characterization [33]. 

**Figure 3:**
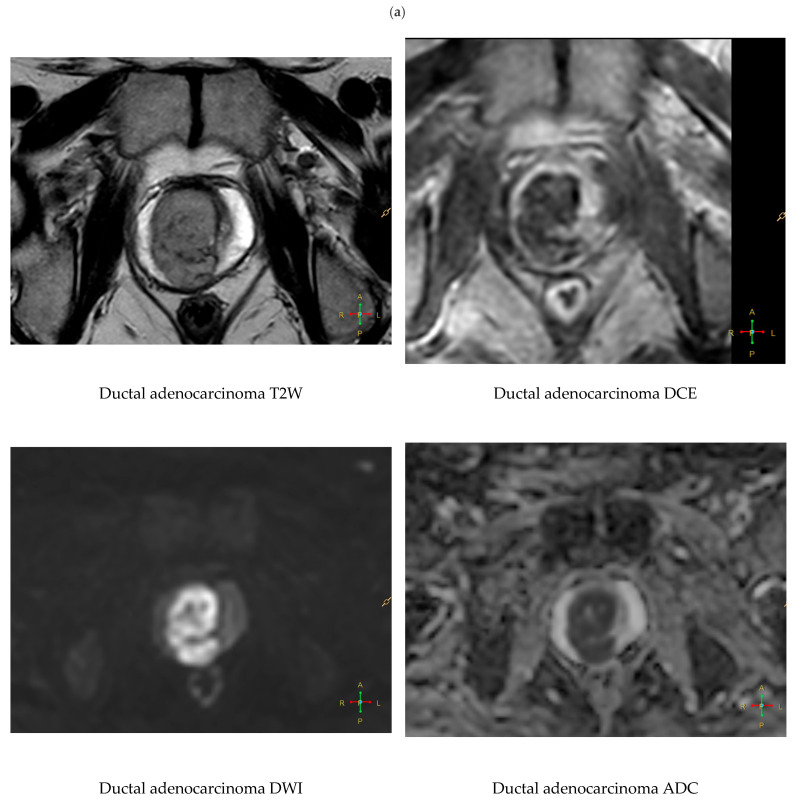

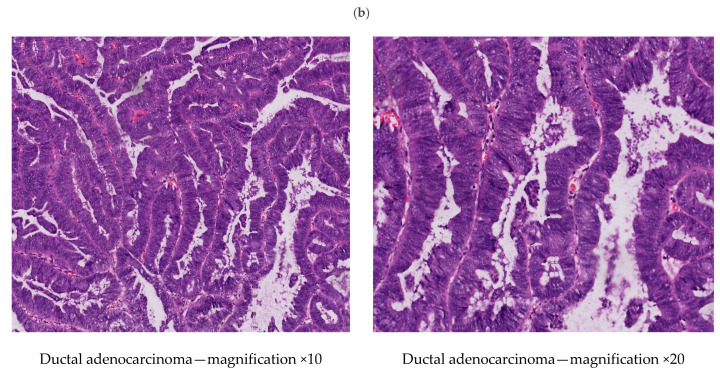
(**a**) Multiparametric MRI of extensive ductal adenocarcinoma located in the transitional zone of the prostate. On axial T2-weighted images, the lesion appears as a large, markedly hypointense and infiltrative mass, effacing the normal zonal anatomy and extending toward the periurethral region. Diffusion-weighted imaging (DWI) demonstrates very high signal intensity with corresponding markedly reduced apparent diffusion coefficient (ADC) values, consistent with severe restriction. On dynamic contrast-enhanced (DCE) imaging, the tumor exhibits strong and early enhancement with heterogeneous internal architecture. These features are characteristic of centrally located ductal adenocarcinoma, which frequently shows aggressive local behavior and a tendency to invade seminal vesicles. (**b**) Histopathological specimen of prostate ductal adenocarcinoma. Papillary and cribriform glandular structures are lined by tall columnar epithelial cells with enlarged nuclei and prominent nucleoli, consistent with ductal morphology. Hematoxylin and eosin (H&E) staining. Representative images at ×10 (**left**) and ×20 (**right**) magnification.

Ductal adenocarcinoma typically arises periurethrally and displays strong early contrast enhancement on DCE and prominent diffusion restriction. It often lacks involvement of the peripheral zone and may be underrepresented in PIRADS-based assessments [34] (Figure 3).

Imaging of basal cell carcinoma is not pathognomonic, but several recurring patterns have been described. On T2-weighted images, the tumor typically appears as a heterogeneous low-to intermediate signal mass in the peripheral or transition zone; in Wang et al., the lesion demonstrated “slightly longer T2” after TURP [12]. On DWI/ADC, diffusion is mildly to clearly restricted, though occasionally subtle [12]. Early enhancement of abnormal soft tissue has been reported pre-treatment—in Toesca et al., abnormal enhancing tissue was observed in the prostate and corpus spongiosum, with complete radiographic response after chemoradiation on follow-up MRI [19]. Extracapsular extension and seminal vesicle invasion have also been documented, mimicking high-grade acinar carcinoma [16]. Metastatic BCC has been reported as well, with pelvic MRI showing no local recurrence despite distant spread to the lung [17]. Collectively, MRI “red flags” include an infiltrative prostate mass with low or heterogeneous T2 signal, diffusion restriction, early enhancement, and disproportionate obstructive symptoms despite low or normal PSA—a clinical clue emphasized across multiple reports [12,16,17].

**Figure 4:**
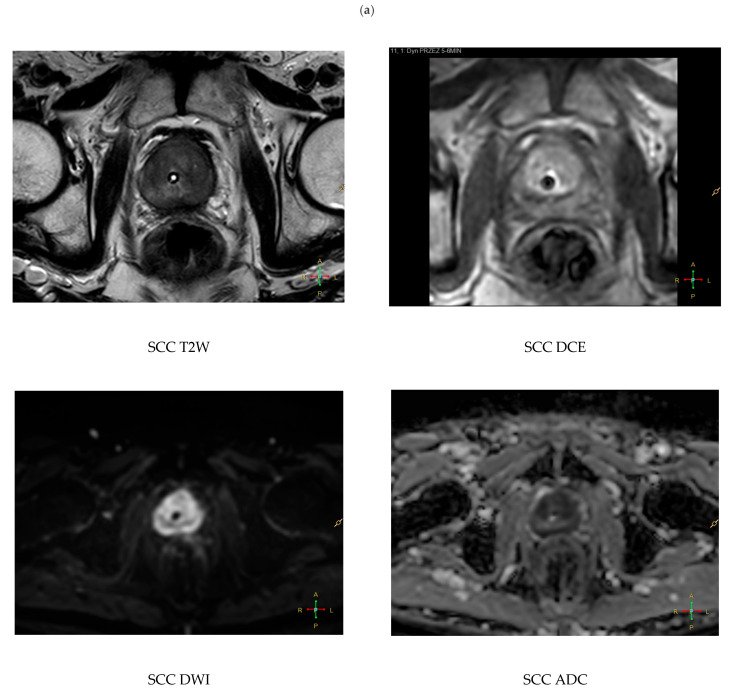

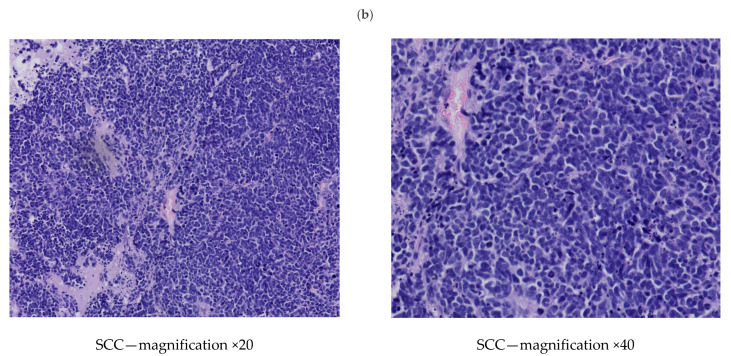
(**a**) Multiparametric MRI of small cell carcinoma of the prostate (SCC) originating from the transition zone (TZ). On axial T2-weighted imaging, the tumor appears as a large, ill-defined hypointense mass with obliteration of the normal zonal anatomy; the Foley catheter is visible within the urethral lumen. Diffusion-weighted imaging (DWI) shows very high signal intensity with markedly reduced apparent diffusion coefficient (ADC) values, reflecting severe diffusion restriction. Dynamic contrast-enhanced (DCE) imaging demonstrates early, heterogeneous enhancement, more pronounced in the TZ compared with the peripheral zone (PZ). These features are consistent with the aggressive neuroendocrine phenotype, often underestimated by standard PIRADS assessment. (**b**) Histopathological specimen of small cell carcinoma of the prostate (SCC), representing the neuroendocrine phenotype. The tumor is composed of sheets of small cells with scant cytoplasm, hyperchromatic nuclei, and finely granular (“salt-and-pepper”) chromatin, accompanied by high mitotic activity, plentiful apoptotic bodies and areas of necrosis. Hematoxylin and eosin (H&E) staining. Representative images at ×20 (**left**) and ×40 (**right**) magnification. The diagnosis was established based on the characteristic small cell morphology despite negative immunohistochemical staining for neuroendocrine markers.

Imaging of primary squamous cell carcinoma, compared with acinar adenocarcinoma, more often shows a solid, exophytic, and locally invasive mass (Figure 4). Hanna et al. described an irregular, solid, enhancing mass with a large exophytic component on axial T2, invading adjacent structures [20]. He et al. reported abnormal prostatic enhancement on MRI in an elderly man with severe lower urinary tract symptoms [21]. When metastatic, PSCC more often produces osteolytic rather than osteoblastic bone lesions, a potentially useful radiologic discriminator from acinar carcinoma [20].

Basaloid and squamous variants typically appear isointense or only mildly hypointense on T2-weighted images and may lack significant diffusion restriction, which increases the risk of false negative interpretations on mpMRI [35].

### 4.2. PSMA and FDG PET/CT

Prostate-specific membrane antigen (PSMA) PET/CT has become the leading molecular imaging modality for staging and restaging of prostate cancer due to its high sensitivity and specificity, particularly in high-grade acinar disease. However, NEPC and small cell carcinoma often show absent or significantly reduced PSMA expression due to downregulation of androgen receptor pathways during neuroendocrine differentiation [5].

In these cases, 18F-FDG PET/CT serves as a superior modality, revealing intense metabolic activity (SUVmax often >10–15) and capturing visceral metastases not seen on PSMA imaging. These findings are particularly relevant for detecting liver, lung, and non-PSMA-avid nodal involvement in NEPC or treatment-emergent small cell carcinoma [4].

Ductal adenocarcinoma often exhibits PSMA avidity, although FDG uptake may be seen in more dedifferentiated forms [36]. Due to limited available data, no specific pattern of FDG uptake has been established. However, in the case reported by Dong et al., strong FDG uptake was observed [37].

High-grade Gleason pattern 5 tumors typically demonstrate intense uptake on both PSMA and FDG PET due to their high tumor cellularity and proliferative index [38].

Emerging PET tracers, including 18F-Fluciclovine and fibroblast activation protein inhibitors (FAPI), are being explored to improve detection in PSMA-negative or dedifferentiated variants; however, current evidence in prostate cancer remains limited and heterogeneous, and FAPI should be regarded as an investigational adjunct rather than a routine tool for aggressive subtypes [39,40].

### 4.3. Bone Scintigraphy

Although still widely used for detecting skeletal metastases, conventional 99mTc-based bone scintigraphy lacks specificity in aggressive variants. While Gleason pattern 5 and ductal adenocarcinoma often produce osteoblastic metastases clearly visualized on bone scans, NEPC and small cell carcinoma more frequently develop osteolytic or mixed lesions, which may be underestimated or missed entirely [41]. Basaloid carcinomas rarely involve bone metastasis but often present as locally advanced disease, with extension into adjacent structures, including the pelvic bones.

Given these limitations, bone scintigraphy should be interpreted in conjunction with cross-sectional imaging and, when appropriate, phenotype-tailored PET/CT for comprehensive metastatic assessment in high-risk patients.

### 4.4. Transrectal Ultrasound (TRUS)

TRUS continues to serve as the primary tool for guiding prostate biopsies, but it is suboptimal for identifying aggressive and centrally located variants. NEPC, small cell, and basaloid tumors often appear isoechoic and may not be detected due to their central gland location and low echogenic contrast [42].

Ductal adenocarcinoma may manifest as a heterogeneous hypoechoic mass in the periurethral or transition zone; however, such findings can mimic benign prostatic hyperplasia and may be underdiagnosed by TRUS-guided biopsy [43]. Likewise, high-grade Gleason tumors in the peripheral zone may appear as irregular hypoechoic foci. Nevertheless, because conventional gray-scale TRUS alone has limited sensitivity and specificity, it should be interpreted in conjunction with mpMRI and, increasingly, PET imaging [44,45] (Table 2).

## 5. Radiomics and Artificial Intelligence in Aggressive Prostate Cancer

Radiomics and artificial intelligence (AI) applied to prostate imaging are increasingly framed as tools for operationalizing tumor aggressiveness rather than as purely descriptive texture analyses. In aggressive prostate cancer phenotypes, radiomics is best positioned when it targets clinically actionable endpoints, such as prediction of extracapsular extension (ECE) and seminal vesicle invasion (SVI), estimation of high-grade disease probability, and quantitative characterization of intratumoral heterogeneity that may reflect dedifferentiation. Recent precision-oncology perspectives further place radiomics within a broader radiogenomics/theragnostics paradigm, in which imaging-derived biomarkers are integrated with molecular and clinical data to support individualized risk stratification and treatment selection [8].

In contemporary prostate imaging research, AI methods have been applied across three main layers of the workflow: automated or semi-automated segmentation, lesion detection/classification, and outcome prediction (e.g., upgrading, ECE/SVI risk, or recurrence-related endpoints). Radiomics-based pipelines typically rely on engineered features with explicit definitions, whereas deep learning approaches can learn representations directly from images; hybrid models combine radiomics features with AI classifiers or fuse deep features with clinical variables. In aggressive phenotypes, the practical rationale for AI-enhanced radiomics is not to replace expert assessment, but to improve consistency in borderline or discordant scenarios (for example, centrally located bulky tumours, atypical PSA kinetics, or suspected dedifferentiation where single- modality readouts are insufficient). Systematic reviews of deep learning–based prostate MRI segmentation report promising accuracy but highlight robustness, domain shift, and external validation as central barriers to routine deployment [46].

Nevertheless, combining radiomics and AI introduces well-recognised trade-offs. Model performance in small and heterogeneous datasets can be inflated by overfitting, and this risk is particularly pronounced for rare histologic variants where sample sizes are limited and endpoints may be inconsistently defined. Cross-centre generalisability is further challenged by protocol variability (scanner, sequence parameters, reconstruction, and post-processing), while segmentation choices can substantially shift feature distributions. For these reasons, the revised manuscript emphasises methodological safeguards—standardised feature definitions (IBSI) and contemporary reporting/quality frameworks (CLEAR, TRIPOD+AI)—as prerequisites for clinical credibility rather than optional best-practice additions [47,48,49].

In prostate MRI, radiomics has been investigated as a supportive biomarker for local staging beyond visual assessment, particularly for ECE prediction. A contemporary systematic review and meta-analysis indicated that MRI-based radiomics models may improve identification of ECE compared with conventional qualitative interpretation, while also demonstrating heterogeneity in feature engineering, segmentation strategies, and validation design across published studies [10]. These limitations are especially relevant in aggressive phenotypes, where robust generalization is required across scanners, protocols, and diverse tumor morphologies.

Radiomics has also been explored for noninvasive grading and risk stratification. A recent systematic review and meta-analysis focusing on MRI-based radiomics for predicting prostate cancer Grade Groups summarized the evolving evidence base and highlighted that performance estimates are sensitive to cohort composition, reference standards, and the choice of validation strategy [11]. In aggressive phenotypes, the pragmatic value of such models lies in supporting decisions when conventional imaging interpretation is challenging or when clinical and imaging features are discordant, rather than in replacing expert assessment.

Beyond MRI, PSMA PET radiomics has become an active area for aggressiveness assessment and staging augmentation, including attempts to refine risk stratification beyond single metrics such as SUVmax. A 2025 review focusing on radiomics/AI applied to staging PSMA PET emphasized potential utility for aggressiveness-related endpoints and for improving consistency of staging decisions, while underscoring that robust multicenter validation and standardization remain the major barriers to routine adoption [9]. In the context of aggressive variants and dedifferentiated states, PSMA PET radiomics may be most informative when interpreted alongside complementary functional information (e.g., FDG PET in selected scenarios) and when the model endpoint is explicitly tied to clinical decision-making.

Importantly, strictly variant-specific radiomics datasets (e.g., dedicated ductal adenocarcinoma or neuroendocrine prostate cancer cohorts) remain limited, and the evidentiary base for dedicated “variant classifiers” is not mature. A defensible translational strategy is therefore to target reproducible imaging correlates of aggressive phenotype and dedifferentiation (e.g., markedly restricted diffusion and heterogeneous lesion architecture on MRI, and, in selected scenarios, discordant PSMA-low/FDG-high functional profiles) and to evaluate performance in clinical endpoints that matter in high-risk care pathways.

Methodological rigor is a prerequisite for clinically credible radiomics. Feature definitions and extraction should follow standardized frameworks such as the Image Biomarker Standardisation Initiative (IBSI), and studies should be reported with transparent quality criteria and modern prediction-model reporting guidance (e.g., CLEAR and TRIPOD+AI) to reduce hidden analytical degrees of freedom, improve reproducibility, and facilitate translation across institutions [47,48,49].

## 6. Discussion

The identification and characterization of aggressive prostate cancer variants carry profound clinical implications. These rare subtypes—including neuroendocrine prostate cancer (NEPC), small cell carcinoma, ductal adenocarcinoma, Gleason pattern 5 tumors, and basaloid carcinomas—differ significantly from conventional acinar adenocarcinoma in terms of morphology, molecular features, PSA kinetics, and imaging behavior. They are frequently underrecognized and may be diagnosed only after progression to advanced or treatment-refractory disease [3,27].

From a diagnostic standpoint, these tumors often present with atypical clinical and radiological features. Low PSA levels, rapid disease progression, unusual imaging localization (e.g., central gland), or lack of PSMA expression should prompt consideration of an aggressive variant [2,12]. Conventional imaging tools such as mpMRI and bone scintigraphy may fail to detect or correctly classify these lesions. Integrating FDG PET/CT and, in some cases, radiomic analysis, may help reveal underlying tumor biology and support subtype differentiation [32,38].

For example, NEPC and small cell carcinoma frequently exhibit intense metabolic activity on FDG PET, while being occult on PSMA-based scans [4,41].

Therapeutically, early recognition of variant histology can dramatically alter treatment planning. NEPC and small cell prostate cancers generally require platinum-based chemotherapy, often in regimens similar to those used in small cell lung cancer [6,50]. Ductal adenocarcinoma may respond to escalated androgen blockade and early radiation [28]. Misclassification of these variants as acinar tumors may lead to suboptimal outcomes, as standard androgen deprivation therapy alone is often insufficient.

Radiomics—a computational approach to analyzing imaging data—holds promise for the non-invasive identification of aggressive phenotypes. In parallel, AI algorithms—ranging from classical machine learning on radiomic features to deep learning representation models—have increasingly been investigated for segmentation, classification, and prediction tasks in prostate imaging workflows. Their potential clinical value in aggressive phenotypes lies primarily in improving reproducibility and integrating multi-modal signals (mpMRI with PSMA/FDG PET and clinical variables), provided that robust external validation and transparent reporting standards are met [46,47,48,49].

Preliminary studies have shown that radiomic features such as entropy, heterogeneity, and shape irregularity may correlate with histologic aggressiveness, particularly in NEPC and ductal tumors [32,34].

While the integration of radiomics into clinical decision-making remains experimental, it represents a step toward more personalized and biologically informed prostate cancer management. Looking ahead, the future of diagnosing and managing aggressive prostate cancer variants depends on a multidisciplinary and data-driven approach. There is a pressing need to integrate molecular and imaging biomarkers, allowing earlier and more accurate subtype classification [6]. Efforts should also focus on the standardization of radiomic feature extraction, as well as imaging acquisition protocols and segmentation methods, to enable reproducibility across centers [51].

In addition, the development of diagnostic algorithms that combine PSA kinetics, imaging features, clinical history, and molecular signatures may enhance diagnostic precision. These efforts must be supported by prospective multicenter trials, which validate radiomic models and assess their utility in clinical workflows [11]. Finally, enhancing awareness among radiologists, urologists, and oncologists through education and updated guidelines will be critical in ensuring these rare but consequential variants are recognized and treated appropriately.

## 7. Conclusions

Aggressive histologic variants of prostate cancer, including neuroendocrine carcinoma, small cell carcinoma, ductal adenocarcinoma, Gleason pattern 5 tumors, and basaloid subtypes, are rare but highly lethal entities characterized by rapid progression, atypical clinical presentation, and resistance to conventional androgen deprivation therapy [1,2,3]. Early and accurate recognition is essential to enable timely subtype-specific treatment [5,6]. Standard diagnostic tools, including PSA testing, transrectal ultrasound, and conventional imaging, frequently fail to adequately characterize these tumors [3,43,52]. Multiparametric MRI, PSMA PET/CT, and FDG PET/CT provide complementary diagnostic information but require careful interpretation, particularly in the setting of discordant PSA kinetics or low PSMA expression [3,28,38]. The PI-RADS v2.1 system, while effective for typical acinar adenocarcinoma, does not explicitly account for variant histologies, potentially leading to underestimation or misclassification [6,38].

Currently, no dedicated diagnostic recommendations specifically address aggressive histologic variants. The ESUR and EAU acknowledge limitations of PSA-based screening and emphasize mpMRI in high-risk disease, while the EAU Guidelines (2024) suggest FDG PET/CT in selected cases with suspected neuroendocrine differentiation or castration-resistant progression [32,33]. Radiomics and quantitative image analysis offer promising tools to improve diagnostic precision and biological characterization, with early evidence linking features such as heterogeneity and shape descriptors to tumor biology and histologic subtype [6,38]. However, methodological heterogeneity and limited external validation remain barriers to clinical implementation [51,53]. An integrated, multidisciplinary approach combining advanced imaging, molecular profiling, and clinical expertise is essential to advance precision diagnostics for these high-risk patients.

## Figures and Tables

**Table 1:** Key Characteristics of Aggressive Prostate Cancer Variants.

Variant	Prevalence (%)	Typical PSA level	PET Visibility	Common Molecular Alterations
**Gleason Pattern 5 Adenocarcinoma**	15–20	High	PSMA+, FDG+	*TP53*, *PTEN*, *SPOP*
**Ductal adenocarcinoma**	0.4–0.8	Moderate to high	PSMA+, FDG+	*ERG*, *TP53*, *SPOP*
**Neuroendocrine prostate cancer**	<2 (de novo); 10–20 (mCRPC)	Low	PSMA−, FDG+++	*TP53*, *RB1*, *MYCN*
**Small cell carcinoma**	<1	Very low	PSMA−, FDG +++	*TP53*, *AURKA*
**Basaloid/squamous carcinoma**	<0.1	Variable	Variable	*TP63*, CK5/6

Abbreviations: PSA—Prostate-Specific Antigen, PSMA—Prostate-Specific Membrane Antigen positron emission tomography (PET) tracer uptake, FDG—[18F]-Fluorodeoxyglucose PET uptake, mCRPC—metastatic Castration-Resistant Prostate Cancer, + indicates uptake; +++ indicates high uptake; − indicates no uptake.

**Table 2:** (**A**) Comparative Imaging Features of Aggressive Prostate Cancer Variants. (**B**) Comparative Clinical Features of Aggressive Prostate Cancer Variants.

(**A**)						
Variant		MRI Features		PSMA PET	FDG PET	Bone Scintigraphy
Neuroendocrine prostate cancer (NEPC)		T2 hypointensity, low ADC, central mass, local invasion		Negative	High SUV (>10–15)	Osteolytic or mixed
Small cell carcinoma		Homogeneous mass, low ADC		Negative	Very high SUV	Osteolytic or mixed
Ductal adenocarcinoma		Central or periurethral location, strong DCE enhancement, restricted diffusion		Positive	Variable (±)	Osteoblastic
Gleason pattern 5 adenocarcinoma		PI-RADS 5 lesion, multiple hypointense foci, low ADC		Strongly positive	Positive	Osteoblastic
Basaloid carcinoma		Iso-to hypointense on T2, minimal diffusion restriction, central location		Low or variable (±)	Low or variable (±)	Mixed or nonspecific
(**B**)						
Variant	TRUS Appearance	PSA Level at Diagnosis	PSA Velocity	PSA Doubling Time (PSADT)	Typical Anatomic Zone	Tumor Size at Diagnosis
Neuroendocrine prostate cancer (NEPC)	Often not visible	Low (<10 ng/mL)	High if rising	<3 months	Central or transitional zone	>3 cm
Small cell carcinoma	Often not visible	Very low	Low	Rapid progression	Central or transitional zone	>4 cm
Ductal adenocarcinoma	Heterogeneous central lesion	High (20–50 ng/mL)	>2–5 ng/mL/year	3–6 months	Transitional or periurethral	2–4 cm
Gleason pattern 5 adenocarcinoma	Irregular hypoechoic foci	Very high (≥50 ng/mL)	>5 ng/mL/year	<3 months	Peripheral zone	>2–3 cm
Basaloid carcinoma	Often not visible	Normal to moderately elevated	0.5–2 ng/mL/year	6–12 months	Central or transitional zone	0.5–2 cm

Abbreviations: ADC—apparent diffusion coefficient; DCE—dynamic contrast enhancement; FDG—[18F]fluorodeoxyglucose; PSMA—prostate-specific membrane antigen; SUV—standardized uptake value.

## Data Availability

The original contributions presented in this study are included in the article. Further inquiries can be directed to the corresponding author.
